# WHSP-Net: A Weakly-Supervised Approach for 3D Hand Shape and Pose Recovery from a Single Depth Image

**DOI:** 10.3390/s19173784

**Published:** 2019-08-31

**Authors:** Jameel Malik, Ahmed Elhayek, Didier Stricker

**Affiliations:** 1 German Research Center for Artificial Intelligence, DFKI, 67663 Kaiserslautern, Germany; 2 Department of Informatics, University of Kaiserslautern, 67653 Kaiserslautern, Germany; 3 School of Electrical Engineering and Computer Science (SEECS), National University of Sciences and Technology (NUST), Islamabad 44000, Pakistan; 4 Computer Science Department, University of Prince Mugrin (UPM), Madinah 20012, Saudi Arabia

**Keywords:** depth sensor, convolutional neural network (CNN), 3D hand pose, 3D hand shape

## Abstract

Hand shape and pose recovery is essential for many computer vision applications such as animation of a personalized hand mesh in a virtual environment. Although there are many hand pose estimation methods, only a few deep learning based algorithms target 3D hand shape and pose from a single RGB or depth image. Jointly estimating hand shape and pose is very challenging because none of the existing real benchmarks provides ground truth hand shape. For this reason, we propose a novel weakly-supervised approach for 3D hand shape and pose recovery (named WHSP-Net) from a single depth image by learning shapes from unlabeled real data and labeled synthetic data. To this end, we propose a novel framework which consists of three novel components. The first is the Convolutional Neural Network (CNN) based deep network which produces 3D joints positions from learned 3D bone vectors using a new layer. The second is a novel shape decoder that recovers dense 3D hand mesh from sparse joints. The third is a novel depth synthesizer which reconstructs 2D depth image from 3D hand mesh. The whole pipeline is fine-tuned in an end-to-end manner. We demonstrate that our approach recovers reasonable hand shapes from real world datasets as well as from live stream of depth camera in real-time. Our algorithm outperforms state-of-the-art methods that output more than the joint positions and shows competitive performance on 3D pose estimation task.

## 1. Introduction

Jointly estimating 3D hand shape and pose is very important for many computer vision (CV) applications such as animation of a personalized hand in virtual reality (VR) and augmented reality (AR), handling objects [1] and in-air signature [2]. This task is very challenging due to various factors including large variation in hand shapes, complex hand poses, many degrees of freedom and occlusions, especially in egocentric viewpoints. CNN-based 3D hand pose estimation from a single depth image has been extensively studied in recent years. Direct hand pose regression methods (discriminative) [3,4,5] show the highest accuracy on public benchmarks. However, these methods do not exploit the hand structure well, which may result in poor estimation of 3D pose on unseen data [6]. On the other hand, structured hand pose estimation methods either implicitly incorporate hand structure [7,8,9] or embed a kinematic hand model in a deep network [10,11,12]. However, the kinematic model parameterization is highly nonlinear, which is difficult to optimize in deep networks [13]. In contrast, we propose a simple and effective structured 3D pose estimation approach that estimates 3D bone vectors using a CNN, which are converted to 3D hand joint positions by a bone-to-joint layer. The novel layer allows resolving the limitations of both discriminative and structured methods as it preserves the hand structure and produces more accurate 3D hand pose because learning bones representation is easier than learning angles of kinematic model [13].

**Figure 1:**
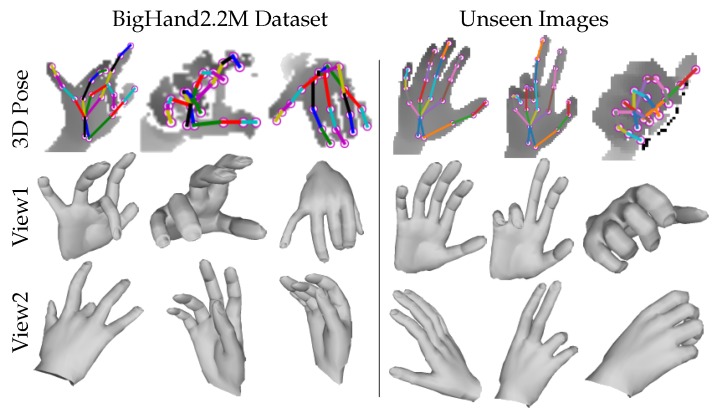
Our proposed approach accurately recovers full 3D hand mesh and 3D pose from a single depth image. We show our results on real dataset as well as on unseen images from real-time demo.

3D hand shape estimation using depth sensors has been studied in [14,15,16,17,18,19,20]. However, these methods employ a generative optimization process which needs a carefully calibrated hand model. On the other hand, deep learning-based simultaneous estimation of 3D hand shape and pose is a novel problem that has not been well investigated yet. This task is highly challenging given the fact that ground truth of real hand shapes is not available. Manual annotation of 3D hand shape is highly time consuming, laborious and sub-optimal. Malik et al. [21] employed the standard linear blend skinning (LBS) function using fixed set of synthetic blendshape targets for hand shape reconstruction which limits this approach to incorporate nonlinear and large variations in hand shapes. Adnane Boukhayma [22] proposed a structured hand shape and pose estimation method from monocular RGB input using the statistical MANO hand model [23]. However, this approach is also limited by a small training data and the LBS based on linear bases. Recently, Ge et al. [24] proposed a weakly-supervised regression based approach that highly depends on a pseudo ground truth of real hand shapes, which is obtained using a pre-trained model with labeled synthetic RGB dataset. Moreover, their 3D pose estimation accuracy directly depends on the quality of real hand shape estimation. In this paper, we propose a novel weakly-supervised algorithm that estimates both 3D hand mesh and pose from a single depth image by learning from unlabeled real data and labeled synthetic data. We argue that learning dense 3D hand mesh from sparse 3D hand joint positions along with a depth synthesizer as a source of weak-supervision is very effective and produces accurate and reasonable hand shapes. We performed rigorous evaluations of our approach on both public real world datasets and a synthetic dataset. Our algorithm can recover accurate and reasonable hand shapes even in cases of missing depth information and occlusion (see Figure 1). To summarize, our contributions for this paper are:

1. A new deep network for structured 3D hand pose estimation embeds a simple bone-to-joint layer to respect hand structure in the learning (see Section 4.1).
2. A novel 3D hand shape decoder generates dense hand mesh vertices given sparse joint positions by mixed training with labeled synthetic data and unlabeled real data (see Section 4.2).
3. A new depth image synthesizer reconstructs 2D depth image from dense 3D hand mesh. It acts as a weak-supervision in training, thereby partly compensating the deficiency of missing hand shape ground truth in real benchmarks (see Section 4.3).
4. A novel weakly-supervised end-to-end pipeline for 3D hand pose and shape recovery, which we call WHSP-Net, is trained by learning from unlabeled real data to a fully-labeled synthetic data (see Section 4).

## 2. Related Work

### 2.1. Depth Based Hand Pose Estimation

A comprehensive review including a detailed comparative analysis of previous depth based hand pose estimation methods can be found in [6,25]. Here, we focus on the closely related works. Regression based methods (e.g., [26,27,28,29,30,31,32,33]) directly estimate 3D joint coordinates or probability density map of joints [4,34] using 2D/3D CNN based networks. Oberweger et al. [30] employed CNNs to refine 3D joints estimations in a feedback loop using an initial pose estimator, a depth image synthesizer and a pose update network. Moon el al. [4] learned 3D heat maps of joints using a voxel-to-voxel network based on a 3D CNN. Rad et al. [3] learned a mapping between synthetic data and the corresponding real data together with the 3D pose. However, these methods may result in geometrically invalid pose estimations, especially on unseen images due to missing structural constraints [6].

Structured hand pose estimation methods (e.g., [7,8,10,11,35,36,37,38,39,40,41,42,43]) include hand structure or a hand model in deep learning. Wan et al. [44] implicitly modeled the joint dependencies by learning 3D offsets in a multi-task cascade network. Zhou el al. [10] embeded a geometric hand model layer inside a deep network. Malik et al. [11] extended this work and learned hand bones scales jointly with 3D pose. Hand model-based approaches mentioned above use joint angles and/or bones lengths based parameterization, which is difficult to optimize in deep networks [13]. In contrast, we propose a simple bone-to-joint layer, which is parameterized by 3D bone vectors. Our approach for 3D hand pose estimation is fully-supervised and respects the structure of the estimated 3D pose.

### 2.2. 3D Hand Shape and Pose Estimation

Deep learning based simultaneous estimation of 3D hand shape and pose is a novel and challenging problem, which has recently attracted an attention of the CV community. Malik et al. [21] proposed a depth-based 3D hand shape and pose estimation algorithm which embeds a nonlinear hand pose and shape model layer inside a deep network. Adnane Boukhayma [22] proposed a similar approach and employed MANO model [23] for shape estimation from monocular RGB image. However, the performance of these methods is limited by small training data and fixed linear bases. Ge et al. [24] regressed 3D hand mesh and pose using a weakly-supervised approach from a monocular RGB input. They estimated hand shape using a Graph CNN, and then regressed the pose from the estimated shape. However, they used pseudo-ground truth of real data, which esd obtained from a pre-trained model on a synthetic dataset. In contrast, we propose a novel weakly-supervised algorithm that effectively learns to reconstruct 3D hand shape from structurally valid estimated 3D pose using a novel hand shape decoder. Our approach learns from an unlabeled real world dataset and a fully-labeled synthetic dataset. Inspired by the authors of [24,45], we leverage a new 2D depth image synthesizer which provides a weak-supervision in training for hand shape and pose estimation.

## 3. Method Overview

**Figure 2:**
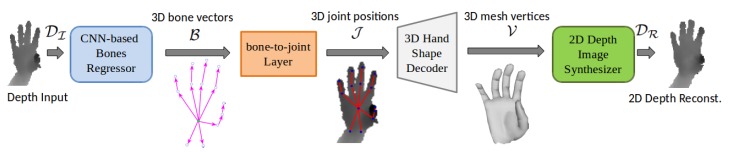
Illustration of our complete method. A hand center cropped depth image $D_{I}$ is provided to a CNN-based bones regressor, which estimates the 3D bone vectors B as an intermediate representation. B is passed to a non-parametric bone-to-joint layer, which converts the 3D bone vectors to 3D joint positions (J). Then, a linear hand shape decoder converts the sparse hand joints positions to dense mesh vertices (V). Finally, a 2D depth image synthesizer reconstructs depth image $D_{R}$ from reconstructed V. The depth synthesizer acts as a weak-supervision in training and is excluded during testing.

In this work, we propose a novel weakly-supervised algorithm that is capable of accurately estimating 3D hand shape and pose using a single depth image. The addressed problem is highly challenging, primarily because there is no ground truth for real hand shapes. In such problems, weak-supervision is an optimal solution. In this regard, Figure 2 shows an overview of our approach. Given a single gray scale cropped depth image $D_{I}$, the task is to estimate 3D hand joint positions $J\in R^{3\times P}$ and 3D hand mesh vertices $V\in R^{3\times N}$ , where P represents the number of joints and N=1193 is the number of mesh vertices. $D_{I}$ is passed to a CNN-based bones regressor, which directly regresses 3D bone vectors $B\in R^{3\times (P-1)}$. A bone $b_{n}\in R^{3}$ is the 3D offset of the *n*th joint ($j_{n}$) relative to its immediate parent joint ($j_{parent(n)}$), and can be calculated as:

(1) $$b_{n}=j_{n}-j_{parent(n)}$$

**Figure 3:**
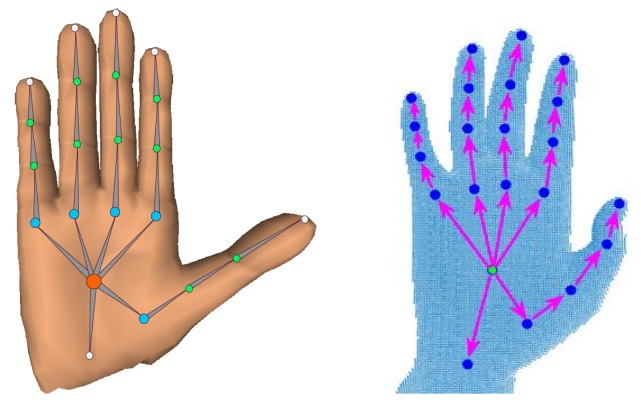
(**left**) SynHand5M [21] dataset hand model; and (**right**) bone vectors and joints of BigHand2.2M [46] dataset hand model.

The direction of bone vector is from parent joint to child joint in the kinematic chain of hand skeleton. as shown in Figure 3 (right). B is an intermediate parametric representation of joints that is fed to a parameter free bone-to-joint layer. This layer allows preserving the structure of hand skeleton (see Section 4.1). For notation simplicity, CNN-based bones regressor and bone-to-joint layer are collectively named as **Module 1**. Thereafter, a linear 3D hand shape decoder (**Module 2**) decodes dense mesh V from sparse pose J (see Section 4.2). In the final stage, a 2D depth synthesizer (**Module 3**) produces a synthesized depth image $D_{R}$ from V (see Section 4.3), which acts as weak-supervision in training. All modules are individually trained and then collectively fine-tuned using mixed synthetic and real datasets (see Section 5). Module 3 is excluded in testing phase.

## 4. The Proposed WHSP Approach

### 4.1. Structured Hand Pose Estimation

In this section, we discuss Module 1 of our pipeline. For better generalized performance, it is important to include hand structure while estimating 3D joint positions [6]. We respect this requirement by introducing a simple bone-to-joint layer, which is embedded inside deep learning. The CNN-based bones regressor estimates intermediate parametric representation B. The CNN architecture is similar to that in [31], which was originally used for directly estimating J. We select this architecture because of its scalability and its highly effective region ensemble (REN) strategy of boosting the accuracy of positions estimation (we refer the reader to [31] for architecture details). Here, we use an ensemble of nine regions and modify the last fully connected (FC) layer to output B. The learning of bones is fully supervised. The bones loss $L_{B}$ is given by the following equation:

(2) $$L_{B}=\frac{1}{2}{∥B-{B_{G}}_{T}∥}^{2}$$

where ${B_{G}}_{T}$ is a vector of ground truth bones.

The proposed bone-to-joint layer is a differentiable and parameter free layer. The task of this layer is to produce J given the estimated B from CNN-based bones regressor. The transformation for one joint $j_{i}$ can be represented as:

(3) $$j_{i}=\left(\prod_{k\in {P_{j}}_{i}}{T_{\phi }}_{k}\left(B_{k}\right)\right){\left[0,0,0,1\right]}^{T}$$

where ${P_{j}}_{i}$ is the set of parent joints of $j_{i}$ in the kinematic chain. T represents a 4x1 translation matrix. $\phi_{k}$ represents the translation along *k*-axis, $B_{k}$ is translational value corresponding to $\phi_{k}$ and [0,0,0,1] is the root joint (i.e., palm center) position. Notably, there are no rotation matrices involved in the transformation since the articulations are represented only by 3D bone vectors. The Euclidean joint locations loss $L_{J}$ is given as:

(4) $$L_{J}=\frac{1}{2}{∥J-{J_{G}}_{T}∥}^{2}$$

where ${J_{G}}_{T}$ is a vector of ground truth joint positions. The gradient computations for the bone-to-joint layer are provided in the Supplementary Materials.

### 4.2. Hand Shape Decoding

**Figure 4:**
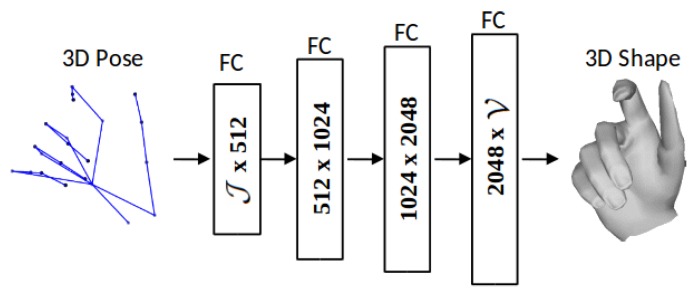
Architecture of the proposed linear 3D hand shape decoder (**Module 2**), which estimates the dense 3D hand mesh representation (shape) from the given sparse 3D joint positions (pose).

As mentioned above, the major bottleneck in 3D hand shape recovery is the missing shape ground truth of real images because manual annotation of real images for shape is a highly time consuming and sub-optimal process. Hence, there is a need to effectively utilize sparse 3D joint annotations in the real datasets in order to learn a reasonable hand shape. In this respect, we propose a novel hand shape decoding method, which is inspired by unsupervised autoencoders [47,48]. The 3D hand pose can be considered as the sparse representation of dense hand mesh. We exploit this inherent relationship between pose and mesh and employ only the decoding part of a linear autoencoder, which maps pose to shape by learning from synthetic data. The architecture of our hand shape decoder is shown in Figure 4. Given the latent pose representation J, the reconstructed mesh V can be represented as:

(5) $$V∼\mathrm{Dec}\left(J\right)=p\left({V_{G}}_{T}|J\right)$$

where $p({V_{G}}_{T}|J)$ is the decoded distribution. The decoder tries to reconstruct V as close as possible to the ground truth ${V_{G}}_{T}$. Both J and V are in the range [−1, 1], therefore *tanh* is used as an activation function after every FC layer. The reconstruction loss $L_{R}$ can be written as:

(6) $$L_{R}=\frac{1}{2}{∥V-{V_{G}}_{T}∥}^{2}$$

The training details with mixed real and synthetic data are presented in Section 5.

### 4.3. Depth Image Synthesis

**Figure 5:**
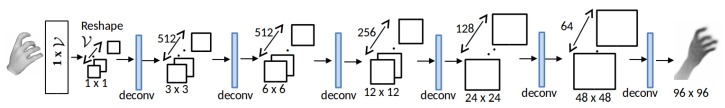
Architecture of the proposed 2D depth image synthesizer (**Module 3**), which is capable of reconstructing a 2D depth image from the given input 3D hand mesh representation by expanding the size of feature maps in both dimensions and finally producing a single gray scale depth frame. **deconv** stands for transposed convolutions.

As discussed above, weak-supervision is an essential component of our pipeline due to the missing shape ground truth of real images. We provide a source of weak-supervision on shape learning by utilizing the input depth image $D_{I}$ and synthesize $D_{R}$ from the reconstructed V, as shown in Figure 2. Inspired by the approaches proposed in [30,45], which synthesize a depth map from sparse joint positions, we build the architecture for our depth image synthesizer to generate depth image from richer dense mesh representation, as shown in Figure 5. It consists of six deconvolution layers, which use *ReLu* as activation functions, except the last layer that uses *tanh*. The sizes of the 2D feature maps increase gradually but decrease in number until $D_{R}$ of size 96×96 is finally synthesized. The kernel sizes for the deconvolution layers are 5×5, 6×6, 9×9, 12×12, 27×27 and 51×51, respectively. We use standard L2 norm to minimize the difference between the synthesized $D_{R}$ and ground truth $D_{I}$ as:

(7) $$L_{D}=\frac{1}{2}{∥D_{R}-D_{I}∥}^{2}$$

The samples of synthesized depth images of NYU [34], BigHand2.2M [46] and SynHand5M [21] datasets are shown in Figure 4 of the Supplementary Materials.

## 5. Network Training

This section gives details about the data preprocessing and training methodology of our complete pipeline. The raw depth images are first hand center cropped based on center of hand mass (CoM). Following Guo et al. [33], CoM is calculated by depth thresholding assuming that hand is the closest object to the camera. For normalization of depth images, the cropping is done along both spatial and depth dimensions using a bounding box of fixed size 150. The final preprocessed image size is 96 × 96 and is normalized in range [−1, 1]. Both joint positions and mesh vertices are made relative to palm center (i.e., CoM) and divided by the bounding box size. After the normalization, all annotations lie in range [−1, 1]. For generalization of the network, we augment training data by applying rotation and scaling in ranges [$-45^{\circ }$, $45^{\circ }$] and [0.8, 1.1], respectively.

After the preprocessing, we train each module of our network individually, and collectively fine-tune them in an end-to-end manner (see Figure 2). We use Caffe [49] for the network training. Module 1 (see Figure 2 in the Supplementary Materials) is trained for jointly optimizing B and J in a fully-supervised manner, using a learning rate (LR) of 0.01 and a batch size of 128. Module 2 (see Figure 4) is jointly trained with real and synthetic datasets, using ground truth annotations pair (J,V) in a semi-supervised manner. Since V is not available for real datasets, we use a simple indicator function layer which implements the following equation:

(8) $$L=𝟙L_{R}$$

where 𝟙 is an indicator function. This layer sends V to the loss layer only for synthetic images using a binary flag value, which is 1 for synthetic and 0 for real. The gradients flow in backward pass is disabled for real data. LR is set to $10^{-4}$ with a batch size of 128. Module 3 (see Figure 5) is individually trained to synthesize $D_{R}$ using only the synthetic dataset because of unavailability of V for real data. The training pair is ground truth (V,$D_{I}$). LR of $10^{-5}$ is used with a batch size of 64. The models run on a desktop PC equipped with Nvidia GeForce GTX 1070 GPU. All networks are trained until convergence. Finally, all modules are put together in a complete pipeline (Figure 2) and fine-tuned on mixed real and synthetic datasets. The overall loss equation of the network can be written as:

(9) $$L_{\mathrm{Full}}=L_{B}+L_{J}+𝟙L_{R}+L_{D}$$

A batch size of 128 is used with an LR of $10^{-7}$ and the full pipeline is trained in an end-to-end manner. Module 3 is excluded during the testing. One forward pass takes only **2.9 ms** to produce both 3D hand mesh and pose.

## 6. Experiments and Results

We performed rigorous evaluation of our method using qualitative and quantitative analysis on both the 3D hand shape and the 3D pose estimation tasks. We provide comparisons with the state-of-the-arts and self-comparisons on both synthetic and real world datasets.

### 6.1. Datasets, Baselines and Evaluation Metrics

None of the existing real hand pose datasets provide ground truth hand shape information. Therefore, we qualitatively evaluated the recovered 3D real hand mesh using two datasets: NYU [34] and BigHand2.2M [46]. NYU provides a train set ($T_{N}$) and a test set, which contain 72,757 and 8252 RGBD images, respectively. The dataset covers a wide range of complex poses but, it is collected from only one subject. It contains 36 annotated joint positions, out of which a subset of 14 joints are used for public comparisons [34]. BigHand2.2M is the largest real dataset, which provides 956 K training depth frames captured from 10 different subjects. The test set for the pose estimation task contains 296 K images. However, the annotations for the test set are not available. Hence, for completeness, we first selected 90% of 956 K (i.e., 860 K) as train set ($T_{B}$) and the remaining frames (i.e., 96 K) as test set. Joint annotations of BigHand2.2M dataset are shown in Figure 3 (right). We manually calculated the hand palm center by taking the mean of the metacarpal joints and the wrist joint. On the other hand, SynHand5M [21] is the largest synthetic hand pose dataset, which contains 5 million depth images with 21 3D joints (see Figure 3, left) and 1193 3D hand mesh vertices as ground truth annotations. Its train set ($T_{S}$) and test set distributions are 4.5 M and 500 K, respectively.

To study the impacts of individual modules on the accuracy of 3D hand pose estimation task, we compared our **Full** model, which is the complete pipeline (see Figure 2), with three baselines. **Baseline 1** directly regresses J (using Module 1 without the bone-to-joint layer). **Baseline 2** is comprised of complete Module 1 while **Baseline 3** constitutes the first two modules of our pipeline (see Section 3). We used four error metrics [21] to evaluate the accuracy of the estimated pose and hand mesh: (i) **3D J Err.**, is the mean 3D joint position error over all test frames; (ii) **3D B Err.** is the average 3D bone location error; (iii) **3D V Err.** gives the mean 3D vertex location error; and (iv) the percentage of success frames within thresholds. All error metrics are reported in mm.

### 6.2. Evaluation of 3D Hand Shape Estimation

This subsection gives the experimental details on 3D hand mesh estimation task using SynHand5M [21], NYU [34] and BigHand2.2M [46] datasets.

**Figure 6:**
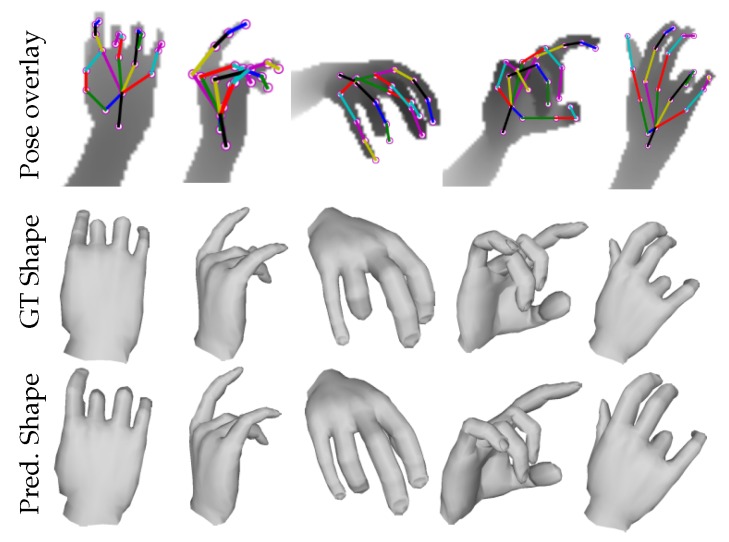
Synthetic hand pose and shape recovery: 3D shape and pose estimation results on SynHand5M [21] dataset.

**Synthetic hand mesh recovery**: As SynHand5M [21] is fully-labeled for pose and shape, we trained Baseline 3 and our Full model in a fully-supervised manner using the training strategy explained in Section 5. Quantitative results are summarized in Table 1. Our Baseline 3 (without using 2D depth image synthesizer) outperforms the state-of-the-art DeepHPS method [21]. Our Full model further improves the accuracy of shape estimation over Baseline 3 by 19.6%. Figure 6 shows the qualitative results on some challenging hand poses of SynHand5M dataset.

**Figure 7:**
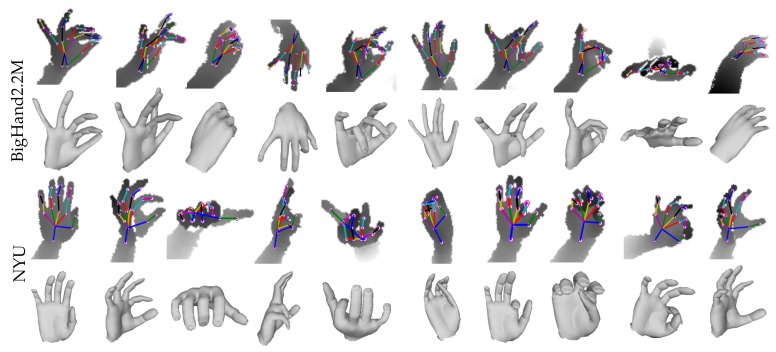
Real hand pose and shape recovery: Our weakly-supervised algorithm is capable of reconstructing accurate and reasonable hand shapes without using any ground truth of hand shapes of real images. We demonstrate the 3D shape and pose estimation results from our proposed method for two real datasets: BigHand2.2M [46] (**top**) and NYU [34] (**bottom**).

**Real hand mesh Recovery**: To effectively learn real hand shapes, Module 3 acts as an important source of weak-supervision in training. To recover the hand shapes of NYU dataset, we combined the train sets of SynHand5M and NYU datasets i.e., $T_{\mathrm{SN}}$ = $T_{S}$ + $T_{N}$, in one unified format and shuffled them. NYU contains a larger set of joint annotations (i.e., 36 joints) than SynHand5M, therefore we selected 16 closely matching joints that are common to both datasets [21]. Our Full model was end-to-end trained on $T_{\mathrm{SN}}$ with total loss of the network given by Equation (9). The mesh loss of Module 2 was computed by implementing the indicator function (Equation (8)). The qualitative results of hand pose and shape recovery on NYU test set are shown in Figure 7. Our algorithm successfully reconstructs reasonable hand shapes of complex poses. Clearly, the quality of shape reconstruction depends on the accuracy of the estimated 3D pose. Examples of synthesized depth images from Module 3 are shown in the Supplementary Materials. Similarly, we jointly trained real BigHand2.2M and synthetic SynHand5M datasets using a mixed train set, i.e., $T_{\mathrm{BS}}$ = $T_{B}$ + $T_{S}$. Both datasets have same annotations, as shown in Figure 3. Qualitative results of BigHand2.2M shapes recovery are shown in Figure 7 and demonstrate successful hand shapes reconstruction even in cases of missing depth information and high occlusions, such as egocentric viewpoint images. More qualitative results from the live stream of depth camera are presented in the Supplementary Materials.

**Figure 8:**
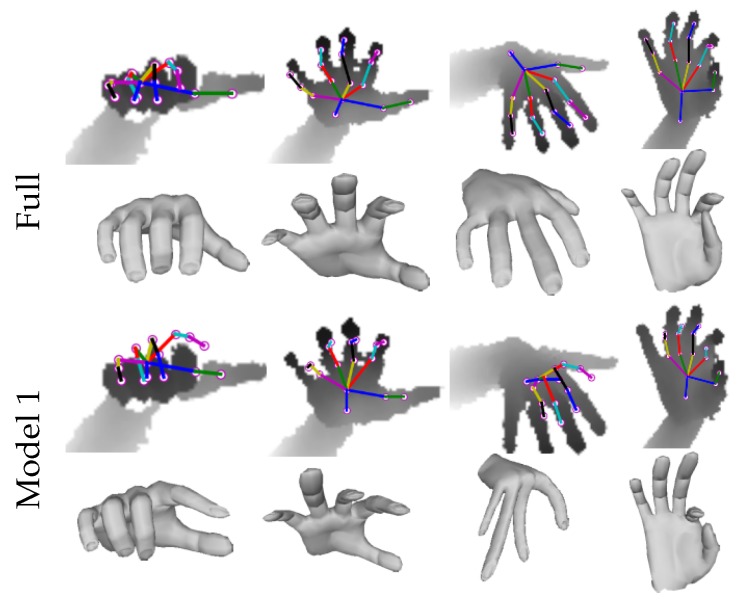
Qualitative comparisons on 3D shape and pose estimation from Full model and Model 1, which clearly show that regressing pose from estimated shape may result in highly inaccurate shape and consequently adverse pose estimation results.

For more rigorous evaluation of our approach for real hand shape recovery, we built a new model, which is inspired by the recent work of Ge et al. [24]. In this model, hand mesh is first estimated using the CNN of Module 1, which directly regresses mesh vertices V from input depth image $D_{I}$, and then a 3D hand pose regressor estimates 3D pose J from the reconstructed V. Finally, the depth image synthesizer synthesizes the depth image $D_{R}$ from J. For notation simplicity, we call this model as **Model 1** and compared its performance with our Full model on NYU dataset (Table 2 shows the pipelines using the notations). Figure 8 shows the qualitative comparison on the sample test images of NYU. Hence, the direct hand shape regression using a single depth image is cumbersome, which may lead to highly inaccurate shape estimation. The pipeline of Model 1 is given in the Supplementary Materials.

**Figure 9:**
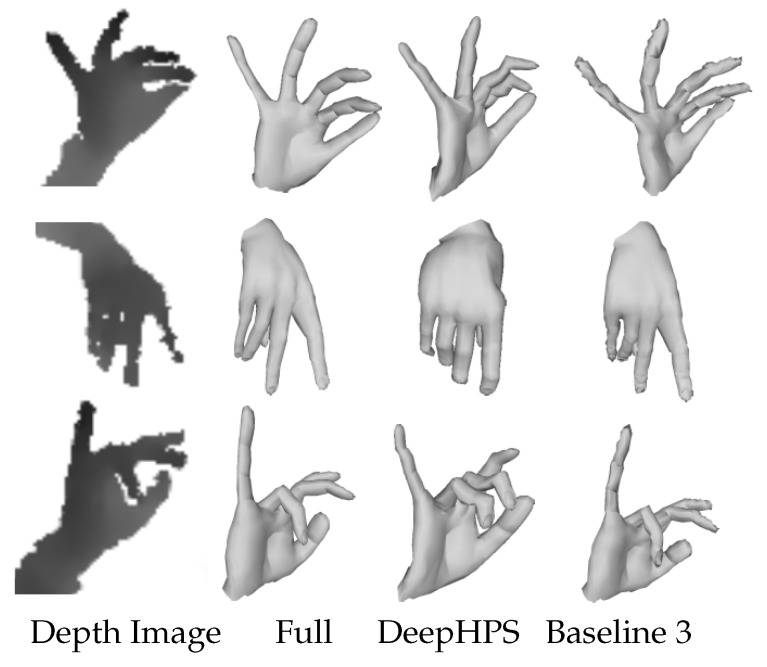
Real hand shape estimation from different methods on **BigHand2.2M** [46]. Our Full model outperforms LBS-based DeepHPS [21] and Baseline 3 (our method without depth synthesizer).

**Comparison with the state-of-the-art**: To qualitatively compare our recovered real hand shape with the state-of-the-art DeepHPS method [21], we implemented this method and trained it on $T_{\mathrm{BS}}$. The results on the sample test images of BigHand2.2M dataset are shown in Figure 9. Artifacts are clearly visible using DeepHPS method due to fixed linear bases (see Section 2) and difficulty in learning complex hand shape and scale parameters in the deep network. In our case, we learn shape from pose, which results in plausible hand shape recovery. We also observed the effect of our Module 3 in training and compared the results of real shape recovery using our Baseline 3. The last column in Figure 9 shows the shape estimation results from Baseline 3, i.e., without using the depth synthesizer. The inaccurate mesh reconstruction with Baseline 3 proves that the addition of a weak-supervision from Module 3 is necessary to get reasonable real hand shape reconstruction.

**Discussion**: Notably, our algorithm learns to reconstruct hand shapes from real depth images by learning from synthetic depth. Therefore, the consistency in depth and joint annotations of real and synthetic images is important to recover the plausible real hand shape and pose. Thus, our approach is unlikely to produce correct and plausible hand shapes for older real hand pose datasets such ICVL [50] and MSRA2015 [51], which are not fully consistent in depth and joint annotations with synthetic SynHand5M [21] dataset.

### 6.3. Evaluation of 3D Hand Pose Estimation

This subsection provides quantitative and qualitative evaluations of our approach on the task of 3D hand pose estimation. We provide self-comparisons and comparisons to the state-of-the-art methods on NYU [34] and SynHand5M [21] datasets. For the sake of completion, we also provide 3D pose estimation results on BigHand2.2M [46] dataset.

**SynHand5M synthetic dataset**: We trained our Baseline 3 and Full model on SynHand5M dataset. The quantitative results for joint positions and bone vectors estimations are provided in Table 1. Our algorithm outperforms the state-of-the-art methods, which shows the effectiveness of our weak-supervised algorithm and its superior performance compared to the state-of-the-art LBS method [21].

**BigHand2.2M real dataset**: We evaluated the accuracy of 3D pose estimation on our created test set from BigHand2.2M dataset [46]. We trained our Full model on mixed train set $T_{\mathrm{BS}}$. Qualitative results are shown in Figure 7, which demonstrate successful 3D pose recovery of complex hand poses even in cases of missing depth and large occlusions. Quantitatively, the 3D joint error on our created test set (see Section 6.1) comes out to be 11.84 mm.

**Figure 10:**
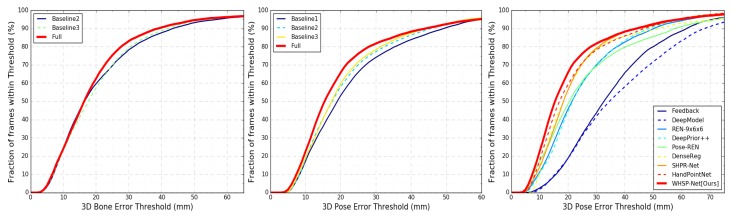
**NYU** [34] dataset: Quantitative results on 3D pose and bone vectors estimation (mm). The curves show the percentage of success frames within certain threshold values: (**left**) comparison of the 3D bone vectors estimation accuracy of Full model with two Baselines; (**middle**) comparison of three Baselines with Full model on joint positions estimation; and (**right**) comparison of our Full model with the state-of-the-art hand pose estimation methods

**Figure 11:**
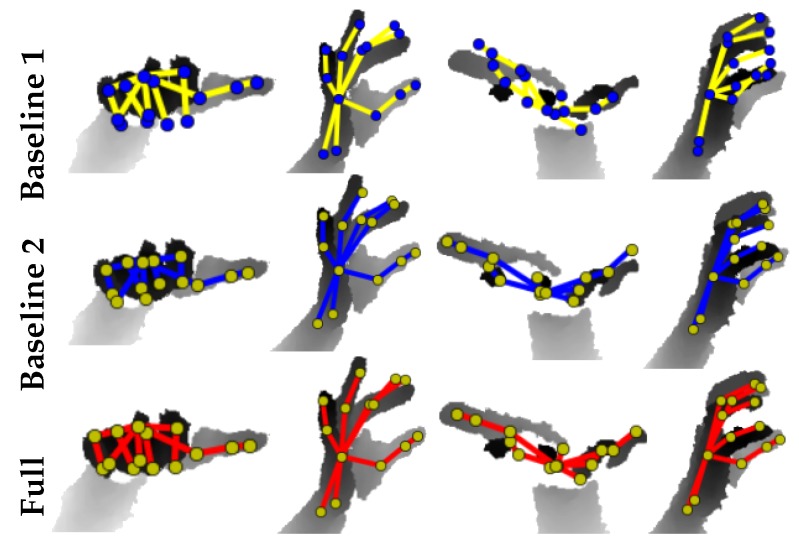
The 3D pose improvement achieved by our Full model compared with the two Baselines on **NYU** [34].

**Self-comparisons**: To rigorously evaluate our algorithm, we performed self-comparisons of our baseline architectures and Full model on real NYU dataset. The networks were jointly trained with combined NYU, BigHand and synthetic SynHand5M datasets and optimized for the loss given by Equation (9). We used the hand model of Zhou et al. [10] for implementing the bone-to-joint layer. Baseline 1 is similar to the CNN architecture proposed in [31], which we use to directly regress J. Table 3 shows the joints estimation accuracy of Baseline 1. Baseline 2, which incorporates hand skeleton structure (see Section 4.1), achieves a 9.6% increase in pose estimation accuracy. Since $L_{B}$ is included in Baseline 2, the 3D bone error is also reported in Table 3. Baseline 3 includes hand mesh learning, which marginally improves the pose estimation accuracy by 2.8% and bones estimation accuracy by 1.9% over Baseline 2. Our Full model shows the best accuracy on joint positions and bone vectors estimations by including Module 3 in training. Figure 10 (left and middle) illustrate quantitative results of the self-comparisons. The curves that cover the most area achieve the highest accuracy. Qualitative comparisons of Baseline 1, Baseline 2 and the Full model are shown in Figure 11. Furthermore, we quantitatively evaluated Model 1 (see Section 6.2), which shows lower accuracy of 3D pose estimation due to inaccurate hand mesh estimation. We compared its performance to our Full model (see Table 2).

**Comparison with the state-of-the-arts**: We compared the 3D hand pose estimation accuracy of our Full model (WHSP-Net) with state-of-the-art approaches. Figure 10 (right) and Table 4 show the quantitative comparisons. Notably, discriminative methods such as V2V-PoseNet [4] and FeatureMapping [3] achieve better accuracy than our method, but they generalize poorly on unseen data [6]. Moreover, V2V-PoseNet is not real-time because of the time consuming gray scale depth input to voxel conversion and the complex 3D-CNN architecture. Furthermore, our method is not discriminative, rather it respects the structure of hand skeleton as well as additionally produces full 3D hand mesh. Therefore, our approach lies in the category of methods that output more than joints. In addition to the 3D pose, DeepModel [10] outputs joint angles; HandScales [11] produces joint angles and bone-lengths; and DeepHPS [21] generates joint angles, bone-lengths, complex shape parameters and full 3D hand shape. Our method outperforms these methods, as shown in Table 4. Our method shows competitive performance to the state-of-the-art methods that do not explicitly consider the hand structure and produce only the 3D pose [3,4,5]. Our algorithm is real-time, producing the 3D pose and shape in 2.9 ms per frame.

## 7. Conclusions

This paper presents a novel weakly-supervised method for a highly challenging problem of 3D hand shape and pose estimation from a single depth image. Our deep network consists of three novel components: (i) Structured 3D hand pose estimator; (ii) 3D hand shape decoder; and (iii) 2D depth image synthesizer. The hand shape decoder learns to recover 3D hand mesh representation from a structurally valid estimated 3D pose. To provide a much needed weak-supervision on shape estimation, we propose a new depth synthesizer which reconstructs 2D depth image from learned hand shape. Our method is jointly fine-tuned on unlabeled real data and labeled synthetic data in an end-to-end manner. Extensive evaluations show plausible and reasonable hand shapes reconstruction in real-time despite an unavailability of ground truth for real hand shapes. The proposed approach outperforms state-of-the-art methods that produce more than joint positions and shows competitive results compared to 3D pose estimation methods.

For future work, we plan to extend our approach using 3D deep networks that establish a one-to-one relationship between an input voxelized depth image [4] and the output 3D hand shape and pose representations.

## Figures and Tables

**Table 1:** Quantitative results on synthetic **SynHand5M** [21] dataset. We compared with the state-of-the-art approaches that produce more than joint positions. Notably, our approach outperforms the recent DeepHPS method, which produces 3D hand pose and 3D shape. All errors are reported in mm.

Method	3D B Err.	3D J Err.	3D V Err.
DeepModel [10]	–	11.36	–
HandScales [11]	6.5	9.67	–
DeepHPS [21]	5.2	6.3	11.8
Baseline 3 [ours]	4.37	5.24	6.37
Full [ours]	**3.71**	**4.32**	**5.12**

**Table 2:** We compared Model 1 with Full model on NYU [34] dataset, which indicates that directly regressing hand mesh from a single depth image is cumbersome and leads to highly inaccurate pose estimation. Mean pose error is in mm.

Method	Pipeline	3D J Err.
Full	$D_{I}$→J→V→$D_{R}$	**10.39**
Model 1	$D_{I}$→V→J→$D_{R}$	23.63

**Table 3:** Self-comparisons on NYU [34] dataset: Yhe effectiveness of different modules of our pipeline. Our Full model shows the effectiveness of jointly fine-tuning the modules altogether. All errors are reported in mm.

Method	3D B Err.	3D J Err.
Baseline 1	–	11.83
Baseline 2	8.40	10.70
Baseline 3	8.24	10.39
Full	**7.80**	**9.24**

**Table 4:** **NYU** [34] dataset: ${}^{*}$ methods that produce more than 3D joints positions; ${}^{+}$ methods that do not respect hand structure and produce only 3D hand pose. WHSP-Net outperforms previous methods that output 3D hand shape and pose, and shows competitive performance to the 3D pose estimation approaches.

Method	3D J Err. (mm)
Feedback [30]	15.9
HandPointNet [7]	10.54
DenseReg [9]	10.214
SHPR-Net [52]	10.77
${}^{+}$MURAUER [5]	9.45
${}^{+}$V2V-PoseNet [4]	8.41
${}^{+}$FeatureMapping [3]	7.44
${}^{*}$DeepModel [10]	17.0
${}^{*}$HandScales [11]	16.0
${}^{*}$DeepHPS [21]	14.20
${}^{*}$WHSP-Net (Ours)	**9.24**

## Supplementary Materials

Samples of the compounds are available online at https://www.mdpi.com/1424-8220/19/17/3784/s1 from the authors.
