# Supplementary material for: WHSP-Net: A Weakly-Supervised Approach for 3D Hand Shape and Pose Recovery from a Single Depth Image
Source: Sensors (Basel). 2019 Aug 31;19(17):3784. doi: 10.3390/s19173784 (PMC6749339; doi:10.3390/s19173784)
Supplement: Supplementary file 1 [file sensors-19-03784-s001.zip › Sensors2019_WHSP_Net_supplementary.pdf]

Article

Supplementary Document for the Paper “WHSP-Net: A Weakly-supervised Approach for 3D Hand Shape and Pose Recovery from a Single Depth Image"

Jameel Malik [1,2,3], Ahmed Elhayek [1,5] and Didier Stricker [1,2]

1 German Research Center for Artificial Intelligence, DFKI, Kaiserslautern 67653, Germany; jameel.malik@dfki.de (J.M.); ahmed.elhayek@dfki.de (A.E.); Didier.Stricker@dfki.de (D.S.)
2 Department of Informatics, University of Kaiserslautern, Kaiserslautern 67653, Germany;
3 School of Electrical Engineering and Computer Science (SEECS), National University of Sciences and Technology (NUST), Islamabad 44000, Pakistan;
4 Computer Science Department,University of Prince Mugrin (UPM), Madinah 20012, Saudi Arabia;
* Correspondence: jameel.malik@dfki.de (J.M.);ahmed.elhayek@dfki.de (A.E.)

Version July 24, 2019 submitted to Sensors; Typeset by LaTeX using class file mdpi.cls

1. Gradients of the bone-to-joint Layer

In this section, we provide the gradient computation details of the bone-to-joint layer which is embedded in the structured 3D hand pose estimator (i.e., Module 1); see Section 4.1 in the paper. For backward-pass in the layer, we compute gradient of the following function (Equation 3 in the paper) with respect to the layer inputs $\mathcal{B}$:

$$j_i = \Big(\prod_{k \in P_{j_i}} \mathrm{T}_{\phi_k}(\mathcal{B}_k)\Big)[0,0,0,1]^{\mathrm{T}} \quad (1)$$

For each joint j_i, the gradient of Equation 1 with respect to a translational value $\mathcal{B}_k$ can be computed as:

$$\frac{\partial j_i}{\partial \mathcal{B}_k} = \Big(\prod_{m \in P_{j_i}} \Gamma_{\phi_m}(\mathcal{B}_m)\Big)[0,0,0,1]^{\mathrm{T}} \quad (2)$$

where,

$$\Gamma_{\phi_m}(\mathcal{B}_m) = \begin{cases} \mathrm{T}_{\phi_m}(\mathcal{B}_m) & \text{if } m \neq k \\ \mathrm{T}_{\phi'_m}(\mathcal{B}_m) & \text{if } m = k \end{cases}$$

and, $\mathrm{T}_{\phi'_m}(\mathcal{B}_m)$ is the derivation of translation matrix with respect to $\mathcal{B}_m$.

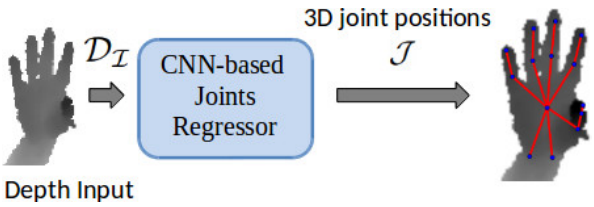

Figure 1. Block diagram of **Baseline 1** network. The preprocessed depth image input $\mathcal{D}_\mathcal{I}$ is passed to a CNN-based joints regressor which directly estimates the 3D joints coordinates $\mathcal{J}$. The CNN architecture is similar to [1].

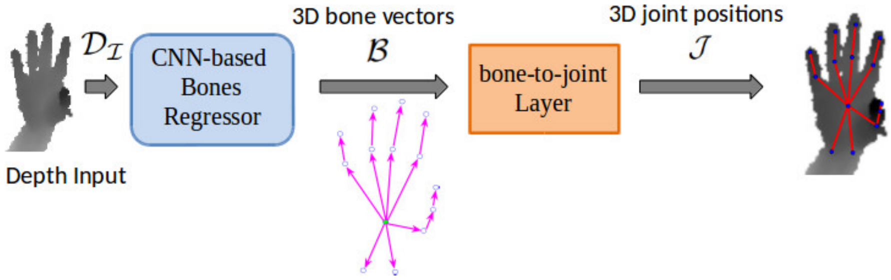

Figure 2. Block diagram of **Baseline 2** deep network. A CNN-based bones regressor estimates 3D bone vectors $\mathcal{B}$ from $\mathcal{D}_\mathcal{I}$ which are sent to a parameter free bone-to-joint layer that produces 3D joint positions $\mathcal{J}$. Learning 3D bone vectors helps to preserve the structure of the hand skeleton in training. The pipeline is trained in an end-to-end manner.

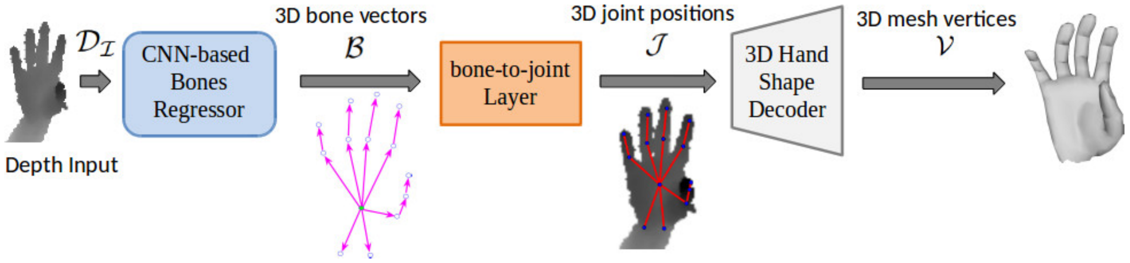

Figure 3. Overview of **Baseline 3** deep network for estimating both 3D hand pose and 3D hand shape. The joint positions $\mathcal{J}$ are estimated from a structured hand pose estimator which consists of the CNN-based bones regressor and the bone-to-joint layer. 3D hand shape decoder linearly decodes hand mesh vertices from joint positions. The complete pipeline is trained in an end-to-end manner.

2. Network Details of the Baselines

In this section, we provide the network details of our **Baseline 1**, **Baseline 2** and **Baseline 3** (described in Section 6.1 in the paper). Figure 1 shows the block diagram of Baseline 1. We leverage the CNN architecture proposed in [1] to directly regress 3D joint positions $\mathcal{J}$. We select this particular architecture because of its scalability and its effective region ensemble strategy which boosts the accuracy of estimated joint positions. The loss equation for Baseline 1 is given as:

$$\mathcal{L}_{\text{Baseline 1}} = \mathcal{L}_\mathcal{J} \tag{3}$$

In Baseline 2, a novel bone-to-joint layer is added to the network of Baseline 1. This addition allows to respect the structure of the hand; see Figure 2. The main advantage of Baseline 3 over other model-based hand pose estimation methods is that the bone vectors are easier to learn than joint angles of kinematic hand model [2]. Therefore, we estimate 3D bone vectors as intermediate representation which are learned by the CNN-based bones regressor. These bone vectors are converted to joint coordinates using Equation 1 by the bone-to-joint layer. The total loss of Baseline 2 is given as:

$$\mathcal{L}_{\text{Baseline 2}} = \mathcal{L}_\mathcal{B} + \mathcal{L}_\mathcal{J} \tag{4}$$

Where $\mathcal{L}_\mathcal{B}$ and $\mathcal{L}_\mathcal{J}$ are the loss terms for bones vectors and joints positions, respectively (see Equations 2 and 4 in the paper). Figure 3 illustrates the pipeline of Baseline3 which estimates both 3D pose and 3D hand shape from a single depth image. The ground truth of hand shapes for real images is not available primarily because annotating such images for shape is time consuming and sub-optimal. Thus, the network of Baseline 3 is trained with mixed real and synthetic data. The total loss can be represented as:

$$\mathcal{L}_{\text{Baseline 3}} = \mathcal{L}_\mathcal{B} + \mathcal{L}_\mathcal{J} + 1\mathcal{L}_\mathcal{R} \tag{5}$$

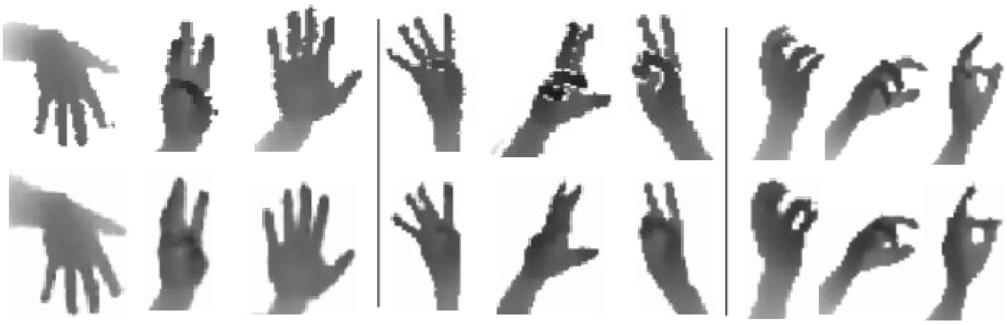

Figure 4. Samples of synthesized 2D depth images of NYU [3](**Left**), BigHand2.2M [5](**Middle**) and SynHand5M [4](**Right**) datasets. **Top:** Ground truth depth frames. **Bottom:** Synthesized depth images from the learned 3D hand shape. The proposed 2D depth image synthesizer reconstructs reasonable depth images and acts as an important source of weak supervision in training.

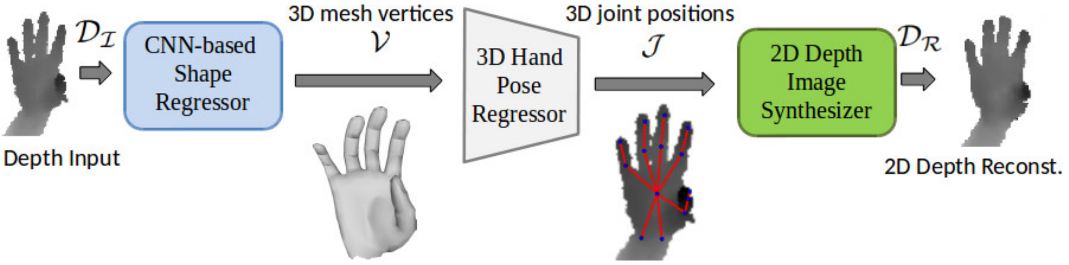

Figure 5. Block diagram of **Model 1** network. The preprocessed depth image $\mathcal{D}_\mathcal{I}$ is passed to the CNN-based hand shape regressor which directly regresses hand mesh vertices $\mathcal{V}$. Then, the 3D hand pose regressor estimates the joints coordinates from the reconstructed $\mathcal{V}$. Finally, the depth image $\mathcal{D}_\mathcal{R}$ is synthesized from the 3D pose $\mathcal{J}$ by the 2D depth image synthesizer.

where $\mathcal{L}_\mathcal{R}$ is the reconstruction loss for 3D hand mesh vertices and 1 is an indicator function. The implementation of 1 was achieved by adding a another new layer to our network. This layer forwards mesh vertices $\mathcal{V}$ to the euclidean loss layer only for synthetic images using a binary flag value which is 1 for synthetic and 0 for real. The gradients flow in backward pass is disabled for real data. However, mixed learning with labeled synthetic and unlabeled real data is not an optimal solution for learning accurate shapes. Therefore in our Full model (see Figure 2 in the paper), we provide a much needed weak-supervision using a 2D depth image synthesizer which learns to reconstructs depth images from estimated shapes.

3. Depth Image Synthesizer Evaluation

In this section, we provide the qualitative results of the proposed 2D depth image synthesizer (see Section 4.3 in the paper). We train our Full model on combined NYU [3] and SynHand5M [4] datasets by employing the network training strategy (please refer to Section 5 in the paper). The qualitative results of samples of reconstructed NYU depth images are shown in Figure 4(Left). We also train our Full model on combined BigHand2.2M [5] and SynHand5M datasets. The sample reconstructed images from BigHand2.2M dataset are shown in Figure 4(Middle). The qualitative results of synthesized depth images of SynHand5M dataset are shown in Figure 4(Right). Our depth synthesizer is able to reconstruct reasonable depth images from estimated hand mesh vertices during end-to-end training with mixed real and synthetic datasets. As the resulting depth images are just an estimate of the input images, they are not useful. However, accurately reconstructing them is an indication that the image synthesizer is doing its job correctly.

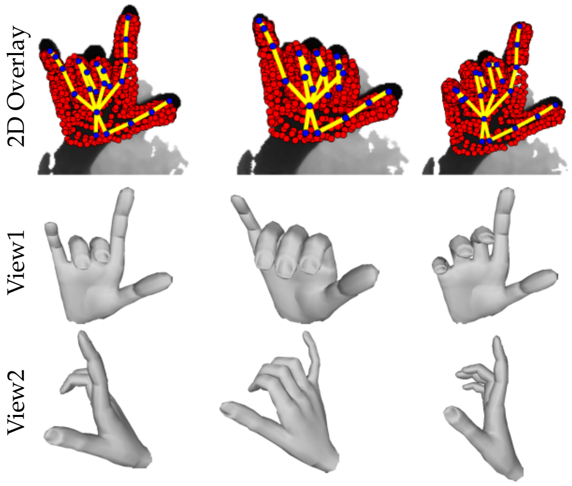

Figure 6. 3D hand mesh and 3D pose recovery from a single depth image. We show our estimation results on unseen images from real-time demo using Creative Senz3D camera.

4. Network Architecture of Model 1

In order to show the effectiveness of our approach, we build a new pipeline (i.e., **Model 1**) which is inspired by the recent work [6] (please refer to Section 6.2 in the paper). The block diagram of Model 1 is shown in Figure 5. 3D hand mesh vertices $\mathcal{V}$ are directly regressed from a single depth image $\mathcal{D}_{\mathcal{I}}$ using a CNN-based shape regressor. The architecture of the CNN is similar to [1] which uses an ensemble of 9 different regions. However, we modify the last fully connected regression layer to output dense mesh $\mathcal{V}$ instead of joint positions. Then, 3D joint positions $\mathcal{J}$ are regressed from the estimated $\mathcal{V}$ using a linear 3D hand pose regressor. Finally, a 2D depth image synthesizer reconstructs 2D depth image from the estimated $\mathcal{J}$. The architecture of the synthesizer is similar to the one shown in Figure 5 of the paper except the first layer takes $\mathcal{J}$ instead of $\mathcal{V}$. The total loss equation of Model 1 is given as:

$$\mathcal{L}_{\text{Model 1}} = 1\mathcal{L}_{\mathcal{R}} + \mathcal{L}_{\mathcal{J}} + \mathcal{L}_{\mathcal{D}} \tag{6}$$

Where $\mathcal{L}_{\mathcal{D}}$ is the reconstruction loss for 2D depth image synthesizer. The qualitative and quantitative comparisons of Model 1 with our Full model are shown in Figure 8 and Table 2 in the paper. The results clearly show that directly regressing real hand mesh vertices from a single depth image is hard and it can lead to inaccurate shape estimation which consequently affects the accuracy of the estimated 3D pose. This fact is explicitly reported in [6] where they have used a pseudo ground truth of hand shapes of real images during fine-tuning with real data, in order to recover reasonable real hand shape using monocular RGB image. Whereas, our approach (Full model) is capable of recovering accurate and reasonable hand shapes without using any pseudo ground truth of real hand shapes (see Section 6.2 in the paper).

5. Results from Live Demo

We train our Full model on mixed real BigHand2.2M [5] and synthetic SynHand5M [4] datasets using the network training strategy explained in Section 5 of the paper. The network is trained till convergence. The system runs in real-time as it completes the forward pass in **2.9ms**. From live stream of Creative Senz3D depth camera [7], we preprocess a raw depth frame using a depth threshold value of 500mm and center of hand mass (CoM) (see Section 5 of the paper). See the **supplementary video** for live demo results. Figure 6 shows the 2D overlay of reconstructed pose and mesh from unseen depth images, and two different viewpoints of the reconstructed shape in 3D. These results show that our approach is capable of successfully recovering reasonable hand shapes and poses in real-time

from unseen real world images. This illustrates that the proposed system is perfect for virtual reality application as it allows the user to see the animation of his hand in the virtual environment.

Bibliography

1. Wang, G.; Chen, X.; Guo, H.; Zhang, C. Region Ensemble Network: Towards Good Practices for Deep 3D Hand Pose Estimation. *Journal of Visual Communication and Image Representation* **2018**.
2. Sun, X.; Shang, J.; Liang, S.; Wei, Y. Compositional human pose regression. The IEEE International Conference on Computer Vision (ICCV), 2017, Vol. 2, p. 7.
3. Tompson, J.; Stein, M.; Lecun, Y.; Perlin, K. Real-time continuous pose recovery of human hands using convolutional networks. *ACM Transactions on Graphics (ToG)* **2014**, *33*, 169.
4. Malik, J.; Elhayek, A.; Nunnari, F.; Varanasi, K.; Tamaddon, K.; Heloir, A.; Stricker, D. DeepHPS: End-to-end Estimation of 3D Hand Pose and Shape by Learning from Synthetic Depth. 2018 International Conference on 3D Vision (3DV). IEEE, 2018, pp. 110–119.
5. Yuan, S.; Ye, Q.; Stenger, B.; Jain, S.; Kim, T.K. Bighand2. 2m benchmark: Hand pose dataset and state of the art analysis. Computer Vision and Pattern Recognition (CVPR), 2017 IEEE Conference on. IEEE, 2017, pp. 2605–2613.
6. Ge, L.; Ren, Z.; Li, Y.; Xue, Z.; Wang, Y.; Cai, J.; Yuan, J. 3D Hand Shape and Pose Estimation from a Single RGB Image. *In CVPR* **2019**.
7. Creative. Senz3D Interactive Gesture Camera. https://us.creative.com/p/web-cameras/creative-senz3d, 2018.

Sample Availability: Samples of the compounds ...... are available from the authors.

© 2019 by the authors. Submitted to *Sensors* for possible open access publication under the terms and conditions of the Creative Commons Attribution license (http://creativecommons.org/licenses/by/4.0/)
